# Application of Meta-Heuristics in 5G Network Slicing: A Systematic Review of the Literature

**DOI:** 10.3390/s22186724

**Published:** 2022-09-06

**Authors:** Rayner Gomes, Dario Vieira, Miguel Franklin de Castro

**Affiliations:** 1 Applied Research to Distributed Systems Lab, Federal University of Piauí (UFPI), Picos 64607-670, Brazil; 2 Efrei Research Lab, EFREI Paris, 97800 Villejuif, France; 3 GREat Research Lab, Federal University of Ceará (UFC), Fortaleza 60455-760, Brazil

**Keywords:** meta-heuristics, network slicing as a service, NSaaS, network slicing, Virtual Network Embedding (VNE), 5G

## Abstract

Network slicing is a vital component of the 5G system to support diverse network scenarios, creating virtual networks (slices) by mapping virtual network requests to real networks. The mapping is an arduous computing process, mathematically studied and known as the Virtual Network Embedding (VNE) problem, and its complexity is NP-Hard. The mapping process is oriented to respect the QoS demands from the virtual network requests and the available resources in the physical-substrate infrastructure. Meta-heuristic approaches are a suitable way to solve the VNE problems because of their capacity to escape from the local optimum and adapt the solution search to complex networks; these abilities are essential in 5G networks scenarios. This article presents a systematic review of meta-heuristics organized by application, development and problem-solving approaches to VNE. It also provides the standard parameters to model the infrastructure and virtual network requests to simulate network slicing as a service. Finally, our work proposes some future research based on the discovered gaps.

## 1. Introduction

Relevant organizations have proposed the concept of network slicing to address the diversified technical service requirements in 5G underpinned by background technologies such as Software-Defined Networking (SDN), Network Function Virtualization (NFV) and Mobile Access Edge Computing (MEC) [1]. The technical service requirements of the vertical sectors are composed of many different values, such as data rate, mobility, latency, reliability, positioning accuracy, and coverage. A network slice is an end-to-end logical network deployed with isolated virtual resources on the shared physical infrastructure. These logical networks are deployed as different services to meet the different communication needs of the user. Therefore, 5G will provide a Network Slicing as a Service (NSaaS) model that can be very flexible in allocating and reallocating resources according to dynamic requirements so that it can adapt network slices for different and complex 5G communication scenarios [2]. The notion of network slicing is to leverage the network infrastructure resources to create multiple sub-networks for different classes of services and applications. Slicing is a crucial component to turning 5G into a reality.

In recent years, regulators and other stakeholders such as research institutions, mobile operators, network equipment suppliers, and international organizations have launched research efforts to adapt the Network Slicing (NS) as omnipresent 5G technology. Some leading groups are the International Telecommunication Union (ITU), European Telecommunications Standards Institute (ETSI), Open Networking Foundation (ONF), 5G Infrastructure Public Private Partnership (5G-PPP), and Next Generation Mobile Network Alliance (NGMN).

The ITU works on the definition of the framework and overall objectives of the future 5G systems, denoted as IMT-2020 systems in ITU terminology [3]. The ETSI has defined several Industry Specification Groups (ISG) to develop standards, and its work [4] has positioned the demands and business requirements beyond 2020 and introduced the network service deployment concept of NS. The ONF, through its work in [5], aims to describe how critical functional aspects of the SDN architecture are applied to enable the business-oriented concept of NS. The 5G-PPP was initiated by the EU Commission and industry manufacturers, telecom operators, service providers, Small and Medium-sized Enterprises (SMEs) and researchers under the name 5G!Pagoda. The 5G!Pagoda aims to align Europe’s and Japan’s views on a 5G slice-based mobile network infrastructure with dynamic creation and runtime management of network slices as a means to deploy private, tailored networks for various mobile services [6]. Finally, the NGMN defines the concept of network slicing as a set of network functions and resources to perform those network functions, forming a complete instantiated logical network to satisfy specific network properties required for end-user service [7].

Some advantages of using NS are: (i) network slicing can provide logical networks with better performance than unit networks; (ii) a network slice can be scaled up or down as the service requirements and the number of users change; (iii) network slices can isolate the network resources of one service from the others and, as a result, the configurations of the different slices do not affect each other; (iv) finally, a network slice is customized according to the service requirements, which can optimize the allocation and utilization of physical network resources [8].

In the 5G system, the NSaaS is a service in which the provider offers the slice creation on-demand. A slice tenant requests a slice by a document named Virtual Network Request (VNR), which contains a virtual network (set of virtual nodes and virtual links) and a set of Quality of Service (QoS) parameters. After receiving a VNR, the provider seeks to meet the requisition by mapping the virtual network to a real network and respecting its QoS demands. This mapping becomes a challenging problem when it seeks to minimize or maximize some objectives. The mapping is a problem known and mathematically named VNE.

The VNE is an NP-hard problem that has been receiving exhaustive attention from researchers, and the works [9,10,11,12] have proved its NP-hardness and its complexity in different ways. The scientific community has been studying the VNE problem extensively. In addition to the notorious advantage, 5G brings new challenges to VNE approaches. For example, the work [13] points out that NS before 5G does not include as many heterogeneous scenarios and inter-domains as in 5G. Traditionally, virtual networks are created by statically allocating the expected network resources, i.e., network, processing/computing, and storage resources. However, this method is often not optimal, as network resources are usually either over-or under-allocated. Technically, developing such a flexible optimal allocation of slice resources is very challenging, especially when considering a set of functional requirements. For this reason, network slicing is a latent issue in computer networks and involves concepts of scheduling, optimizing, and predicting resource usage. Moreover, looking for future network generations, this problem becomes harder due to the complex scenarios in the sense of more world computing and networking integration [14].

In this work, we present a review of 18 papers to answer some questions considering the application of meta-heuristics to solve VNE problems in 5G scenarios: (i) What is the standard network structure to evaluate the Virtual Network Embedding Approaches (VNEA)? (ii) What is the standard method and structure to create a set of vnr? (iii) What are the most used solver and meta-heuristics to deal with VNE in the literature? (iv) What are the most demanded QoS parameters? (v) What measures do the works use to evaluate mapping approaches? (vi) What types of nodes do the papers use to simulate the network infrastructure?

The remainder of this paper is organized as follows: Section 2 presents our systematic review methodology; Section 3 reveals the results after grouping the works and extracting the information; Section 4 relates the final considerations and suggests future research.

## 2. Methodology

**Figure 1:**
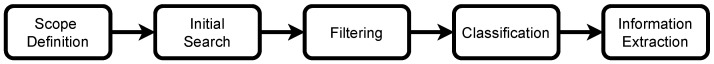
Stages of systematic review.

The systematic review methodology adopted in this work is based on the study of Biolchini et al. [15]. As depicted in Figure 1, the process consists of five stages: definition of scope, initial search, filtering, classification, and information extraction. Regarding the scope, we developed questions to conduct the searches, while in conducting the search, strategies were outlined. In the end, we present the results.

### 2.1. Scope Definition

The first phase is dedicated to the definition of the scope. In this phase, the research questions are essentially developed. These questions limit the scope of the work and, later, the search terms to select the work. Thus, the starting point of this work is to formulate these eight questions in order to delimit the scope of the work:

- Q1: What are the main categories that the approaches fall into? Fischer’s taxonomy [9] defines 6 classification possibilities, and through this question, we want to know in which classes the approaches are.
- Q2: What forms of coordination of the mapping of nodes and links do the approaches use? Coordination indicates the order of the node and link mapping process. For example, the process can perform the mapping of nodes first and then the links, or vice versa. The third way is without any coordination; that is, the mapping of nodes and links can coincide without one process interfering.
- Q3: What are the meta-heuristics applied in the mapping process? The literature is rich in meta-heuristics; through this question, we want to know which ones are used in the works.
- Q4: Are there parallelized versions? For this question, we want to know if there are parallelized versions and how parallelization is used to solve mapping problems.
- Q5:What are the main evaluation metrics? The reason for this question is to find out what are the main evaluation criteria of algorithms.
- Q6: Are approaches aware of multi-domains and network services? Naturally, the 5G network is composed of heterogeneity of technologies and composed of shared infrastructure; furthermore, through the use of the Network Function Virtualization paradigm, services can be spread across the network, so the purpose of this question is to find out if mapping approaches explore these two characteristics of the 5G system.
- Q7: What are the required parameters and what are their values to simulate the network slicing service? The purpose of this question is to find out how the physical infrastructure and virtual networks are modeled.
- Q8: Considering a 5G system, which QoS parameters are part of the requests? With this question, we want to find out, in addition to the traditional CPU and bandwidth demands, if the works are considering the following QoS demands in the requests: reliability, mobility, energy savings, location, wavelength, and security.

### 2.2. Initial Search

Cao et al. [16] classified the VNE approach into three categories: exact solution, heuristic and meta-heuristic. The authors mentioned that the exact solution is limited to small networks and that the meta-heuristic solution aims to find a suitable solution with low execution time when faced with realistic network scenarios. Furthermore, exact approaches can easily suffer from the problem of becoming stuck in local optimal solutions that may be far from the global optimum with respect to optimization theory; on the other hand, the meta-heuristic solution can improve the quality of results by moving away from the local optimum and can be more easily applied in different domains. Thus, our work is interested in works that use meta-heuristics to solve VNE problems.

**Figure 2:**
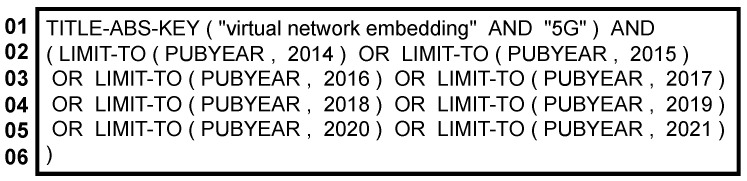
Search string.

Therefore, the second stage addresses Initial Search, and it contemplates the creation of the search terms (Figure 2). Many scientific-paper searchers are available; however, the Scopus (www.scopus.com, accessed on 10 January 2022) and Web of Science (mjl.clarivate.com/home, accessed on 10 January 2022) indexer are the two main searchers. Stahlschmidt and Stephen [17] indicate that these two indexers index reputed journals. However, there is a massive intersection between their results, so there is no reason to use both. Based on their study, the Scopus indexer has a slightly more significant number of articles and reviews published related to the computer and information science field. For that reason, we chose Scopus as the unique indexer. Figure 2 shows the text string inserted in the Scopus; after receiving the results, the filtering stage began.

In addition, we included the work of Fischer et al. [9], which is a cornerstone in the virtual network embedding literature; the authors selected 78 references on virtual network embedding approaches. One of the significant contributions is the creation of a new taxonomy in which articles can be classified as: (a) whether the solution is centralized or distributed; (b) whether the requests are static or dynamic; (c) if the approaches are concise or redundant. Although this work is a comprehensive and well-referenced survey, we did not find the answer to our questions in that article (Section 2.1); thus, we aggregate the selected works from [9] to our results captured in the initial survey.

### 2.3. Filtering

**Figure 3:**
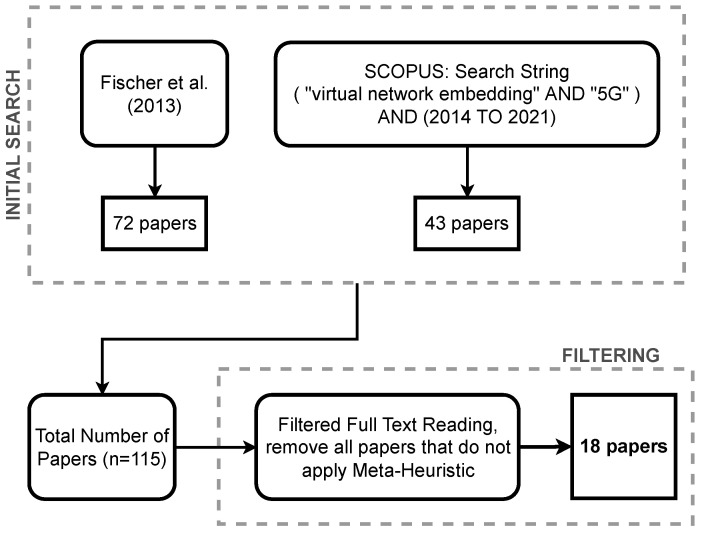
The methodological process comprises the Fischer et al. [9] work and our search, ending with the filtering flowchart.

The third stage deals with Filtering. The filtering strategy was based on the exclusion of works that do not apply meta-heuristics to solve the virtual network embedding problem; therefore, this criterion limits the results inside the scope of our research. Figure 3 shows that, initially, we had 115 articles obtained from [9] and our search. After a straightforward filtering processing, consisting of excluding all documents that do not use meta-heuristics, the final number of articles analyzed was 18.

### 2.4. Classification and Information Extraction

The fourth stage deals with Classification. The classification consists of grouping the remained papers in the groups to allow us to count the number of documents related to (a) category of solutions; (b) types of coordination; (c) the primary solvers’ approach; (d) parallel or not parallel approaches; (e) the main competitors; (f) the main virtual network embedding measurements; (g) multi-domain; (h) infrastructure and virtual network requisitions modeling features; (i) the main QoS parameters demanded and openings.

After the classification process, the following stage is the Information Extraction. In this last stage, the papers were counted and organized. For each group, we presented a summary, and that report is shown in the next section (Section 3).

## 3. Results

Table 1 summarizes the number of works classified in [‘C’/‘D’]/[‘S’/‘D’]/[‘C’/‘R’]. Fischer et al. defined that taxonomy is a tuple formatted by three characters separated by two slashes. The first characters can be ‘C’ for solution centralized or ‘D’ for distributed. The second character can be ‘S’ for the static solution; the algorithm fixes the number of requisitions or value equal to ‘D’ for the dynamic quantity of requisitions, in which requisitions arrive at a certain rate. The third part can be ‘C’ for concise, which means that solutions do not have any reliability mechanism, and the value ‘R’ is for solutions with mechanisms of enhancing the reliability. Table 1 reveals that the recent works have intensified distribution approaches, and in addition, the majority of works are centralized solutions.

Coordination refers to the mapping process types that the VNE approach can achieve. They are: (a) one stage: the selection of nodes and links are made together; (b) two stages: the selection is carried out in two stages; first, the VNE approach maps the node and then the link, or vice versa; (c) uncoordinated: the selection of nodes and links are performed at the same time without one interfering with the other. Table 2 shows that most jobs are two-stage, but single-stage jobs tend to be faster.

Table 3 presents the most common solvers in the papers, which are Ant Colony optimization algorithms (AC) [18]; Genetic Algorithm (GA) [19,20,21,22,23,24,25], Greedy [26,27], Markov Random Walk (MRW) [28], Particle Swarm Optimization (PSO) [27,29,30,31,32], and Simulated Annealing (SA) [33]. The sum of the solvers is greater than the number of articles as in the work [27], which uses two approaches, one using Greedy and the other using PSO.

Table 4 shows how parallel solutions have obtained more attention. Our survey filters only work in the 5G scenarios, and these works need to improve the computing capacities to face the complexity of recent slicing constraints. In this same way, distributed solutions obtained more attention in recent works.

The Greedy, D-VINE and R-VINE (Table 5) approaches stand out as the leading competitors. Greedy is a simple approach; it generally has the shortest execution time. There are many variations in valuing the best choice of the node or link, but despite the different forms of valuation, the algorithm always chooses the best option at each step. R-VINE is a random node mapping-based approach with the shortest path method for link mapping, and D-VINE is based on a deterministic node mapping with the shortest path method for link mapping. In favor of these approaches, even in recent research, both algorithms often provide excellent performance when implementing a relaxed linear programming approach to node mapping.

Table 6 presents the virtual network embedding measurements. Virtual Network Acceptance Rate (VNAR) denotes the number of virtual networks mapped with success. Stress is a measure that indicates the distribution of mapping along the network, and lesser values mean better distribution. There are many other measurements, some of them specific to evaluate each approach; however, from the perspective of the network provider, the Runtime and VNAR are more important due to their association with provider profit. The sum of the values in the table is greater than 18 because the use of metrics is not exclusive, that is, some works use more than one metric at the same time.

There are VNE works that consider the concept of multi-domain and others do not; the majority, 16 works, do not consider this concept (see Table 7). There are two ways of dealing with domains, one way divides the network into technological sections (e.g., [30]), and the other divides the network into administrative areas (e.g., [32]). The technical sections separate the computer network into distinct areas whose sites have different characteristics, and an example is the division of the network into access, transport, and core. The administrative domain considers that parts of the network have an administrator who independently applies policies, e.g., pricing, traffic classification, security, and admission control. Furthermore, the mapping process may consider that the request requires some service associated with the slice; only the paper [31] has this ability.

As mentioned before, some works consider the technology domain to select the nodes; in this case, the physical infrastructure is segmented into areas; the distinct regions of the 5G network are access, transport, and core. In practice, these areas are distinguished by their transmission capabilities, delay, reliability, and access heterogeneity. For example, Table 8 reveals that only one work considers access and core networks.

Table 9 presents the main tools found to assist the work simulations. The primary tool used in the papers is the Georgia Tech Internet Topology Model (GT-ITM) [40]; it is a network topology generator used to create flat random and hierarchical graphs to represent network substrate and virtual network requisitions. Other works use their framework to create datasets to represent the substrate and requisitions; the advantage of this way is that the frameworks are more problem-oriented. Only one work uses the Mininet software [41]. This software is adequate for creating a realistic virtual network with the real kernel, switch, and application code on a single machine with a single command.

Each virtual network request has a lifetime; for the sake of abstraction, most works use the concept of Time Unit (TU). Around 61% of the jobs use the exponential distribution to select a value for the lifetime of the requests; in this modality, 100% of all jobs choose between 0 and 1000 TU. Only three jobs create requests with fixed values. Four papers could not capture the values used in the duration of the requests (Table 10).

All selected works consider that requests arrive over time. Fischer et al. [9] define this mode as dynamic. Dynamic VNEs are the most realistic as the network allocation service will serve slices over time. Most works use the arrival rate as a Poisson distribution (Table 11); the average value of the rate is five requests per 100 TU. Only four of the eighteen articles selected did not report the request creation rate.

Table 12 reveals that the Poisson distribution and fixed values are the most used to define the number of nodes. The real nodes represent the physical nodes that make part of the infrastructure, and the most common formation is oriented to use fixed values; generally, the number of nodes is between 8 and 100. Considering the virtual nodes, the works typically use a uniform distribution, and the number of virtual nodes is between 2 and 20. Two results did not inform how to generate the number of virtual nodes.

Similar to the nodes’ formation, the simulation tools use fixed or dynamic values to choose the number of links. The fixed mode is the most common for creating connections in the real physical model, and the probability way is more common for selecting the number of virtual links in the virtual network models. In Table 13, the Probability and Waxmax labels present the dynamic forms. Through the probability method, the algorithm takes all pairs of possible nodes and randomly chooses whether the couples will have a connection or not; the most common probability value is 50%. With a probability of 50%, the average of the links can be calculated with the equation n·(n−1)/4, where *n* indicates the number of nodes. The Waxman random graph model places *n* uniformly random nodes in a rectangular domain. An edge joins each pair of nodes at a Euclidean distance *d* with probability alpha and beta, and the most common value of alpha is 0.5, and beta is 0.2.

Table 14 describes some resources assigned to nodes and links. The most common resource is the CPU for nodes and bandwidth for connections. Virtual network embedding approaches use these features to constrain the mapping process. For example, only one job considers the disk resource, and another considers the link delay. The critical point is that no work considered the existence of memory (RAM).

The QoS parameters in Table 15 point out the essential aspects in the definition of different types of slices in 5G; they were pointed out by the 5G Architecture Working Group as part of the 5G PPP Initiative [1] with the characteristics that are present in the 5G architecture that will allow the definition of the network slices, mainly concerning enhanced mobile broadband (eMBB), massive machine-type communications (mMTC), and ultra-reliable and low-latency communications (URLLC).

The location is associated with the slice’s feature to contemplate the end-to-end concept of a virtual network. The virtual network request can delineate the geographic areas the slice must cover; only work [27] incorporates this property. Wavelength is a demand in works that consider the aspect of network access to 5G that occurs through wireless networks. In this case, the slicing system can protect the slices from overlapping waves avoiding collision; only work [26] incorporates this property. Reliability is essential for URLL slices and is present in supporting dense networks of IoT. It is a primary enabler for many unique use cases in manufacturing, power transmission, transportation, and healthcare; only one work [38] considers this property.

No work considered mobility, energy efficiency, and security in the demands of virtual network requests. Energy efficiency is defined as the duration of time for a component to be operational without a power supply or is related to energy consumption. Mobility implies if the mapping algorithm considers that mobile devices can move between different access areas over time; in this case, the mapping system must allocate extra resources based on the locomotion prediction to guarantee the permanence of the connection. Finally, despite being one of the most sensitive aspects nowadays due to the profusion of personal data on networks, no work has explored security.

## 4. Final Considerations and Future Works

We have investigated the use of meta-heuristics to solve virtual network embedding problems. First, we extracted data from two sets of articles; the first set was taken from the corresponding document published by Fischer et al. [9]. We then started the second group through a systematic process limited to the domain of 5G, VNE, and meta-heuristics. Our interest is because meta-heuristics use their schemes to escape the local optimum, and they are suitable for complex networks, in addition to the fact that each meta-heuristic has gone through a scientific review process. For these reasons, we have studied the application of meta-heuristics to the VNE problem.

We have asked several questions that are not systematically answered in the current literature; therefore, we have classified and organized the responses and presented the rejoinders in numerous tables to facilitate reading. The results will enable researchers to develop simulation environments for network slicing considering 5G.

The scope of CPU and bandwidth are the ubiquitous demands on the requests. However, regarding a 5G network, we have found that reliability, mobility, energy savings, location, and security are poorly explored. Moreover, at least bandwidth, delay, and reliability are expected to be considered simultaneously in 5G works. In real scenarios, the values of QoS parameters and their priorities may vary; thus, the new approaches must be aware of them simultaneously.

One technique to deal with scalability is parallelism, which is poorly explored in the literature. Our survey found these recent works [21,22,25,39] from 2019 to 2020 that use parallelisms. However, the parallelism technique is not incorporated into the meta-heuristic. The authors’ solution is derived from the updated genetic algorithm with parallel preprocessing to reduce the runtime. However, parallel preprocessing builds a collection of paths for each virtual link, called a path pool, based on its source and destination pairs. The path pool is used by n instances of the genetic algorithm to perform the mapping. It is only possible in small networks, and the meta-heuristic remains sequential.

Meta-heuristics is a large and flourishing field that can be applied naturally to various problems. We found the following meta-heuristics in the works: AC, GA, Greedy, MRW, PSO, and SA. In this comprehensive work [42], the authors Arıcı and Kaya evaluated six well-known population-based optimization algorithms (Artificial Algae Algorithm (AAA), artificial bee colony algorithm (ABC), Differential Evolution (DE), Genetic Algorithm (GA), gravitational search algorithm (GSA) and Particle Swarm Optimization (PSO)). Their work was conducted on the CEC’17 test functions using these algorithms and comparing their performance. The evaluations showed that AAA was the most successful among these six. Therefore, for future work, we propose the application of aaa to VNE problems, taking into account the aforementioned QoS parameters that were not presented in the works.

## Figures and Tables

**Table 1:** Category of solutions.

Year	C/S/C	C/S/R	D/D/C	C/D/C
2011	1	2	0	0
2012	2	3	0	0
2017	1	0	0	0
2019	1	0	2	0
2020	1	0	3	2
Total	6	5	5	2

**Table 2:** Coordination.

One Stage	Two Stages	Uncoordinated
4	13	1

**Table 3:** The main solver approaches in VNE.

AC	GA	Greedy	MRW	PSO	SA
1	9	2	2	5	1

**Table 4:** Quantity of parallel and non-parallel works.

Year	Parallelized	Quantity
2011	Yes	0
2011	No	3
2012	Yes	0
2012	No	5
2017	Yes	0
2017	No	1
2019	Yes	2
2019	No	1
2020	Yes	3
2020	No	3

**Table 5:** The main competitors.

Competitors	Example	Quantity
VNE-Least	[34]	1
VNE-Cluster	[34]	1
GLPK	[35]	1
PSO	[36]	1
Own	[24,28,29]	3
R-VINE	[37]	5
D-VINE	[37]	6
VNE-Greedy	[34]	11

**Table 6:** Virtual network embedding approach measurements.

Acceptance Ratio	Runtime	Stress
11	6	5

**Table 7:** Multi-domain and service approach.

No-Multidomain	Multidomain	Adm-Multidomain	No-Service	Service
16	1	1	17	1

**Table 8:** Types of nodes.

Reference	Access	Transport	Core
[28]	No	No	No
[18]	Yes	No	Yes
[29]	No	No	No
[26]	No	No	No
[27]	No	No	No
[33]	No	No	No
[19]	No	No	No
[20]	No	No	No
[21]	No	No	No
[38]	No	No	No
[22]	No	No	No
[39]	No	No	No
[30]	No	No	No
[31]	No	No	No
[23]	No	No	No
[24]	No	No	No
[25]	No	No	No
[32]	No	No	No

**Table 9:** Tools to assist the VNE process.

GT-IMT	Own	Mininet
12	5	1

**Table 10:** Types and duration of requests.

Type	0–500 TU	0–1000 TU	Quantity
Exponentially distributed	5	6	11
Fixed	2	1	3

**Table 11:** Quantity of virtual network requisitions.

Type	0–500	1000–4000
Poisson	2	9
Fixed	2	1

**Table 12:** Node and virtual node formation and quantity.

	Uniform Distributed	Fixed	Minimal Quantity	Maximum Quantity
Real Nodes	1	17	8	100
Virtual Nodes	13	3	2	20

**Table 13:** Link and virtual link formation and quantity.

	Fixed	Probability	Waxman	0–500	500–3000
Real link	5	4	5	11	3
Virtual link	2	10	3	14	0

**Table 14:** Most common resources associated with nodes and links.

	Uniformly Distributed	Fixed	0–50	50–100	100–3000	Not Informed	Not Used
CPU	15	1	2	13	1	2	0
vCPU	14	0	4	14	0	4	0
Bandwidth	14	1	1	12	2	3	0
vBandwidth	14	0	14	0	0	4	0
Disk	1	0	0	1	0	0	17
vDisk	1	0	0	0	0	0	17
Delay	1	1	1	1	0	0	16
vDelay	1	1	1	0	0	0	16

**Table 15:** Main 5G network slices required parameters.

Reference	Reliability	Mobility	Energy Saving	Location	Wavelength	Security
[28]	No	No	No	No	No	No
[18]	No	No	No	No	No	No
[29]	No	No	No	No	No	No
[26]	No	No	No	No	Yes	No
[27]	No	No	No	Yes	No	No
[33]	No	No	No	No	No	No
[19]	No	No	No	No	No	No
[20]	No	No	No	No	No	No
[21]	No	No	No	No	No	No
[38]	Yes	No	No	No	No	No
[22]	No	No	No	No	No	No
[39]	No	No	No	No	No	No
[30]	No	No	No	No	No	No
[31]	No	No	No	No	No	No
[23]	No	No	No	No	No	No
[24]	No	No	No	No	No	No
[25]	No	No	No	No	No	No
[32]	No	No	No	No	No	No
